# Maternal urogenital schistosomiasis; monitoring disease morbidity by simple reagent strips

**DOI:** 10.1371/journal.pone.0187433

**Published:** 2017-11-01

**Authors:** Oyetunde T. Oyeyemi, Alexander B. Odaibo

**Affiliations:** 1 Department of Biological Sciences, University of Medical Sciences, Ondo, Ondo State, Nigeria; 2 Department of Zoology, University of Ibadan, Ibadan, Oyo State, Nigeria; George Washington University School of Medicine and Health Sciences, UNITED STATES

## Abstract

**Background:**

Urine analysis is one of the recommended antenatal guidelines for early diagnosis of pregnancy-associated complications. While in practice, urine analysis by dipstick had been used to provide useful information on other urinary tract infections, its applications for early detection of urogenital schistosomiasis in pregnant women is often times not given due attention in most endemic areas. Our study therefore assessed the performance of some common urinalysis parameters in the diagnosis of maternal urogenital schistosomiasis in endemic rural communities of Nigeria.

**Methodology/Principal findings:**

The cross-sectional epidemiologic survey of urogenital schistosomiasis was conducted among pregnant women in Yewa North Local Government, Ogun State, Nigeria. The women were microscopically examined for infection with *Schistosoma haematobium*, visually observed for macrohematuria, and screened for microhematuria and proteinuria using standard urine chemical reagent strips. Of 261 volunteered participants, 19.9% tested positive for *S*. *haematobium* infection. The proportion of microhematuria (23.8%) was significantly higher than that of macrohematuria (3.8%) and proteinuria (16.8%) (*P*<0.05). Microhematuria with sensitivity (82.7%) and specificity (89.0%) was the best diagnostic indicator of urogenital schistosomiasis. Macrohematuria with the least sensitivity (11.8%) was however the most specific (98.1%) for diagnosing urogenital schistosomiasis in pregnant women. Maximum microhematuria sensitivity (100.0%) was observed in women between 15–19 years but sensitivity was consistently low in older age groups. Maximum sensitivity, specificity and predictive values (100.0%) were recorded for microhematuria in first trimester women. Diagnostic efficiency of proteinuria and macrohematuria was also better in the first trimester women except the 25.0% specificity recorded for proteinuria. The overall diagnostic performance of microhematuria and proteinuria was better in secundigravidae.

**Conclusions/Significance:**

Microhematuria can be used for early detection of urogenital schistosomiasis in endemic areas especially in younger women. However because microhematuria is a condition that occurs during pregnancy and in several other diseases, it is necessary to compliment the diagnosis with other diagnostic tools such as microscopy and serology. Treatment with praziquantel is recommended for the women in their late trimesters after follow up test in order to avert associated adverse pregnancy outcomes.

## Introduction

Schistosomiasis is a public health problem in low resource poor countries of the world due to lack of access to health facilities and safe water, poor hygiene and sanitation. The disease is caused by *Schistosoma* spp. *Schistosma mansoni* and *S*. *haematobium* are the two predominant species in sub-Saharan Africa causing human disease. The earlier causes intestinal and hepatic schistosomiasis while the later which is thought to affect the urinary and genital organs causes urogenital schistosomiasis. With the over 260 million infected people in the world [1] and about 97% of all schistosomiasis infections arising from African continent [2], prompt diagnosis for rational control is necessary. Although it is a disease often associated with children [3–6], studies have shown that other population groups are also at risk of *Schistosoma* infection [7,8,9]. Pregnant women in most endemic areas of Africa are at risk due to involvement in domestic work that requires unsafe water exposure. The last decade estimated population of infected pregnant women totalled 10 million [10]. With increasing reports on *Schistosoma* infection during pregnancy [9,11–15], management of infected pregnant women through treatment with praziquantel in late trimesters has been advocated in order to avert undesirable pregnancy outcomes [16]. Despite recommendations from WHO, many endemic countries are reluctant to include it because of the side effects [10]. Besides pregnancy associated morbidities, female genital involvement associated with *S*. *haematobium* infection characterized by symptoms like pelvic discomfort, vaginal discharge, sandy patches in mucosa, edema, and contact bleeding [17] could be presented in non-pregnant and pregnant women alike.

The choice of a rapid diagnostic test is dependent on the following; affordability, sensitivity, specificity, user-friendliness, rapidity and robustness, equipment free and deliverability to end-users [18]. Presently, the only commercially available rapid immunodiagnostic kit for schistosomiasis, the lateral flow urine-CCA dipstick showed very poor performance with the detection of *S*. *haematobium* [19,20], while the lateral flow urine-CAA dipstick originally designed for *S*. *haematobium* is not yet available for public use. This is further supported by a meta-analysis of different studies in Africa on performance of dipstick diagnosis of *S*. *haematobium* [21,22].

Urine analysis is one of the recommended antenatal screenings for early diagnosis of pregnancy-associated complications. In practice, urine analysis by dipstick had been used to provide useful information on other urinary tract infections such as cystitis and urethritis, but its applications for early detection of maternal urogenital schistosomiasis is often times down played in most endemic areas. There are several reports on performance of simple reagent strips for the diagnosis of urogenital schistosomiasis in many sub-Sahara African countries [4–7,23]. Many studies on diagnostic performance of reagent strips in the general population usually included women of reproductive age, although few are specifically focus on this group [24,25], while no study, to the best of our knowledge, has been specifically conducted on pregnant women. Prompt monitoring of the disease in pregnant women through the use of avoidable and easy to use chemical reagent dipsticks in low resource endemic rural areas becomes very imperative for effective management and to avert the often schistosomiasis-associated undesirable pregnancy outcomes such as low birth weight, prenatal and maternal death [26].

Hematuria is a recognized as pathological condition of urogenital schistosomiasis and its detection using reagent strips had long been suggested as a simple and rapid means of screening large populations [27,28]. While proteinuria is also a useful indirect diagnostic indicator of urogenital schistosomiasis, its application during pregnancy may be undermined by some common abnormal pregnancy conditions such as preeclampsia. These biomarkers of infection due to *S*. *haematobium* are known to correlate with the intensity of infection [29]. Our study therefore assessed the performance of these urinalysis parameters in the diagnosis of maternal urogenital schistosomiasis in endemic rural communities of Nigeria.

## Materials and methods

### Ethical statement

Permission was sought from the communities’ leaders prior study. Communities’ heads and primary health care workers were contacted in advance of the survey to ensure maximum participation of the pregnant women. Written informed consent was obtained from the women. Study was voluntary and participants were permitted to opt out at any time. Ethical approval (UI/EC/11/0052) was obtained from the joint ethical review committee of University College Hospital and University of Ibadan, Ibadan, Nigeria. Infected pregnant women in second and third trimesters were administered a single dose of 40 mg/kg praziquantel [10]. The first trimester women were treated at the later stage of their pregnancy.

### Study area

The study was conducted in Yewa North Local Government Area (LGA). It forms part of the epidemiological studies on urogenital schistosomiasis conducted among pregnant women between February 1, 2010 and February 15, 2011 [9]. As previously described in several studies carried out in the study area, transmission of schistosomiasis is aided by lack of potable water supply and presence of snails intermediate hosts of schistosomes [9,15,29–31].

### Study design and sample size determination

The study was descriptive and cross-sectional. Participants were recruited according to an already described procedure in our previous study [9]. Briefly, the LGA was divided into 10 wards based on the presence of functional primary health care centers. Subjects were then sourced from these health centers using a convenient random sampling. Written informed consents were obtained from the participants. Non-regular dwellers were excluded from the study. A minimum sample size 223 as determined by the method of Daniel [32] using a 0.05 (5%) precision and 80% statistical power was computed. A 30% prevalence of urogenital schistosomiasis obtained from a total of 30 pregnant women randomly selected in a pilot study was also used for sample size determination [9].

### Rapid diagnostic tests and parasitological examination

Collection and examination of urine samples was done according to previously described procedures [6,9]. Fresh mid-day single urine sample each collected from 261 participants by trained personnel in a pre-labeled sterile universal bottle between the hour of 10 and 14 o’clock was inspected for gross hematuria (macrohematuria) and then screened for microhematuria and proteinuria using urine reagent strip (Medi-Test Combi 9^®^, Neumann-Neander-Str. 6–8. D-52355 Düren). The urinalysis was performed according to the manufacturer’s instructions. The diagnostic performance of the morbidity indicators of urogenital schistosomiasis was determined. The samples were transported to the Parasitology Research Unit Laboratory of the Department of Zoology, University of Ibadan for further analysis. The sample was mixed and 10 mL of the sample was centrifuged. The sediment was observed under the light microscope after removal of supernatant for the presence of terminally spined *S*. *haematobium* eggs.

### Statistical analysis

The data were entered into an Excel spread sheet (version 2007) and transferred to GraphPad Prism 5 (GraphPad Software, Inc., La Jolla, CA, USA) for analyses. Data were categorized into age group (15–19, 20–24, 25–29, ≥ 30 years), trimester and parity. Chi square and Fisher’s exact tests were used to determine the significant differences in proportion of urogenital schistosomiasis in subjects positive and negative for pathological indicators of the disease. Diagnostic performances of reagent strips in different categories of data were determined by calculating sensitivity, specificity, positive and negative predictive values and areas under the receiver operating characteristic (ROC) curves. Urine microscopy was used as the gold standard. *P*-values < 0.05 were considered statistically significant.

## Results

### Associations between schistosomiasis and morbidity indicators in pregnant women

The distribution of urogenital schistosomiasis and morbidity indicators of the disease in the observed subjects relative to age, trimester and gravidity was presented in Table 1. Infection due to *S*. *haematobium* was associated with macrohematuria and microhematuria (*P* <0.05) (Table 2). While infection statuses were significantly higher in microhematuria positive group compared with those without microhematuria (*P* <0.0001) across age groups, no significant variations were recorded for infection statuses in macrohematuria and proteinuria across different groups (*P* >0.05). The association between urogenital schistosomiasis and indicators of infection stratified by age, trimester and gravidity is presented in Table 3.

**Table 1:** Population characteristics and infection statuses by microscopy and urinary indicators of urogenital schistosomiasis.

Variables		No. examined	Total	Mic	Macro	Micro	Protein
			GMI	No. positive (%)	No. positive (%)	No. positive (%)	No. positive (%)
Age (year)	15–19	30	29.8	3 (10.0)	1 (3.3)	4 (13.3)	4 (13.3)
	20–24	74	25.6	21 (28.4)	6 (8.1)	26 (35.1)	21 (28.4)
	25–29	71	15.6	13 (18.3)	1 (1.4)	13 (18.3)	5 (7.0)
	≥ 30	86	12.1	15 (17.4)	2 (2.3)	19 (22.1)	14 (16.3)
		261	18.4	52 (19.9)	10 (3.8)	62 (23.8)	44 (16.8)
Trimester	1^st^	6	27.7	2 (33.3)	1 (16.7)	2 (33.3)	5 (83.3)
	2^nd^	74	30.8	11 (14.9)	6 (8.1)	15 (20.3)	15 (20.3)
	3^rd^	181	15.5	39 (21.5)	4 (2.2)	45 (24.9)	24 (13.3)
Gravidity	Primi	93	30.7	21 (22.6)	5 (5.4)	24 (25.8)	19 (20.4)
	Secund	49	9.7	12 (24.5)	2 (4.1)	12 (24.5)	7 (14.3)
	Multi	119	15.6	19 (16.0)	3 (2.5)	26 (21.8)	18 (15.1)

GMI; geometric mean intensity (eggs per 10 mL urine), Mic; microscope, Macro; macrohematuria, Micro; microhematuria.

**Table 2:** Association between urogenital schistosomiasis and indicators of infection in pregnant women.

Indicators		No. examined	Infection status		*P values*
			Positive (%)	Negative (%)	
Macrohematuria	Positive	10	6 (60.0)	4 (40.0)	0.005
	Negative	251	46 (18.3)	205 (81.7)	
Microhematuria	Positive	62	41 (66.1)	21 (33.9)	< 0.0001
	Negative	199	11 (5.5)	188 (94.5)	
Proteinuria	Positive	44	14 (31.8)	30 (68.2)	0.039
	Negative	217	38 (17.5)	179 (82.5)	

Note Chi-square analysis was used to determine association between morbidity indicators and *S*. *haematobium* infection statuses in pregnant women.

**Table 3:** Association between urogenital schistosomiasis and indicators of infection stratified by age, trimester and gravidity.

				Infection status		*P values*
Indicators	Variables	Categories	No. examined	Positive (%)	Negative (%)	
Macrohematuria	Age (years)	15–19	1	1 (100.0)	0 (0.0)	0.100
		20–24	6	3 (50.0)	3 (50.0)	0.343
		25–29	1	0 (0.0)	1 (100.0)	1.000
		≥30	2	2 (100.0)	0 (0.0)	0.029
	Trimester	1^st^	1	1 (100.0)	0 (0.0)	0.333
		2^nd^	6	3 (50.0)	3 (50.0)	0.343
		3^rd^	4	2 (50.0)	2 (50.0)	0.203
	Gravidity	Primi	5	3 (60.0)	2 (40.0)	0.074
		Secund	2	1 (50.0)	1 (50.0)	0.434
		Multi	3	2 (66.7)	1 (33.3)	0.066
Microhematuria		15–19	4	3 (75.0)	1 (25.0)	0.001
		20–24	26	18 (69.2)	8 (30.8)	< 0.0001
		25–29	13	9 (69.2)	4 (30.8)	< 0.0001
		≥30	19	11 (57.9)	8 (42.1)	< 0.0001
	Trimester	1^st^	2	2 (100.0)	0 (0.0)	0.029
		2^nd^	15	10 (66.7)	5 (33.3)	< 0.0001
		3^rd^	45	29 (64.4)	16 (35.6)	< 0.0001
	Gravidity	Primi	24	18 (75.0)	6 (25.0)	< 0.0001
		Secund	12	9 (75.0)	3 (25.0)	< 0.0001
		Multi	25	13 (52.0)	12 (48.0)	< 0.0001
Proteinuria		15–19	4	1 (25.0)	3 (75.0)	0.360
		20–24	21	7 (33.3)	14 (66.7)	0.577
		25–29	5	1 (20.0)	4 (80.0)	1.000
		≥30	14	5 (35.7)	9 (64.3)	0.063
	Trimester	1^st^	5	2 (40.0)	3 (60.0)	1.000
		2^nd^	15	4 (26.7)	11 (73.3)	0.218
		3^rd^	23	7 (30.4)	16 (69.6)	0.282
	Gravidity	Primi	19	5 (26.3)	14 (73.7)	0.759
		Secund	7	4 (57.1)	3 (42.9)	0.051
		Multi	17	5 (29.4)	12 (70.6)	0.333

Note Chi-square analysis was used to determine associations between *S*. *haematobium* infection and indicators of infection stratified by age, trimester and gravidity. Where n is < 5, Fisher’s exact test was used to determine associations.

### Diagnostic performance and pregnant women age

The diagnostic performance of dipsticks relative to the age of pregnant women is presented in Table 4. Maximum microhematuria sensitivity (100.0%) was observed in women between 15–19 years but sensitivity was consistently low in other older age groups. Sensitivity of the tested diagnostic indicators seemed to decrease with increase in age of the pregnant women except in women ≥ 30 years (Table 5). Maximum specificity and positive predictive values (100%) were recorded for macrohematuria in pregnant women in age 15–19 years. While the sensitivity of proteinuria was consistently higher than that of macrohematuria across different age groups, the reverse was the case for specificity (Table 5).

**Table 4:** Performance of urinary morbidity indicators in diagnosis of urogenital schistosomiasis in pregnant women.

Diagnostic parameters	Macrohematuria	Microhematuria	Proteinuria
Sensitivity (SS)	11.5 (4.4–23.4)	82.7 (69.7–91.8)	26.9 (15.6–41.0)
Specificity (SP)	98.1 (95.2–99.5)	89.0 (84.0–92.9)	85.7 (80.2–90.1)
Positive predictive value (PPV)	60.0 (26.2–87.8)	65.2 (52.4–76.5)	31.8 (18.6–47.6)
Negative predictive value (NPV)	81.7 (76.3–86.3)	95.4 (91.4–97.9)	82.5 (76.8–87.3)
Odd ratio (OR)	6.7 (1.8–24.7)	38.6 (16.7–89.4)	2.2 (1.1–4.5)
ROC of area under curve (AUG)	0.6(0.5–0.6)	0.9(0.8–0.9)	0.6(0.5–0.7)

*Note*: ROC; receiver operating characteristics.

**Table 5:** Effect of pregnant women age on diagnostic potential of urinary indicators of urogenital schistosomiasis.

Diagnostic	Age groups	Diagnostic indicators (%)		
parameters	(years)	Macrohematuria	Microhematuria	Proteinuria
SS	15–19	33.3 (0.8–90.6)	100.0 (29.2–100.0)	33.3 (0.8–90.6)
	20–24	14.3 (3.0–36.3)	85.7 (63.7–97.0)	33.3 (14.6–57.0)
	25–29	0.0 (0.0–0.2)	69.2 (38.6–90.9)	7.7 (0.2–36.0)
	≥ 30	13.3 (1.7–40.5)	73.3 (44.9–92.2)	33.3 (11.8–61.6)
SP	15–19	100.0 (87.2–100.0)	96.3 (81.0–99.9)	88.9 (70.8–97.7)
	20–24	94.3 (84.3–98.8)	84.9 (72.4–93.3)	73.6 (59.7–84.7)
	25–29	98.3 (90.8–100.0)	93.1 (83.3–98.1)	93.1 (83.3–98.1)
	≥ 30	100.0 (94.9–100.0)	88.7 (79.0–95.0)	87.3 (77.3–94.0)
PPV	15–19	100.0 (2.5–100.0)	75.0 (19.4–99.4)	25.0 (0.6–80.6)
	20–24	50.0 (11.8–88.2)	69.2 (48.2–85.7)	33.3 (14.6–57.0)
	25–29	0.0 (0.0–97.5)	69.2 (38.6–90.9)	20.0 (0.5–71.6)
	≥ 30	100.0 (15.8–100.0)	57.9 (33.5–79.8)	35.7 (12.8–64.9)
NPV	15–19	93.1 (77.2–99.2)	100.0 (86.8–100.0)	92.3 (74.9–99.1)
	20–24	73.5 (61.4–83.5)	93.8 (82.8–98.7)	73.6 (59.7–84.7)
	25–29	81.4 (7.0–90.0)	93.1 (83.3–98.1)	81.8 (70.4–90.2)
	≥ 30	84.5 (75.0–91.5)	94.0 (85.4–98.4)	86.1 (75.9–93.1)
*OR*	15–19	33.0 (0.8–90.6)	123.7 (4.2–3669.0)	4.0 (0.3–58.6)
	20–24	2.8 (0.5–15.0)	33.8 (8.0–141.8)	1.4 (0.5–4.2)
	25–29	1.4 (0.05–36.8)	30.4 (6.4–143.9)	1.1 (0.1–11.0)
	≥ 30	26.5 (1.2–583.3)	21.7 (5.6–84.4)	3.4 (1.0–12.4)

### Diagnostic performance and trimesters

Maximum sensitivity, specificity and predictive values (100.0%) were recorded for microhematuria in first trimester women. Diagnostic efficiency of proteinuria and macrohematuria was also better in the first trimester women except the 25.0% specificity recorded for proteinuria (Table 6). The highest sensitivity (90.0%) was recorded in secundigravidae.

**Table 6:** Effect of gestational age on diagnostic potential of urinary indicators of urogenital schistosomiasis.

Diagnostic	Trimester	Diagnostic indicators (%)		
Parameters		Macrohematuria	Microhematuria	Proteinuria
SS	1^st^	50.0 (12.6–98.7)	100.0 (15.8–100.0)	100.0 (15.8–100.0)
	2^nd^	27.3 (6.0–61.0)	91.0 (58.7–100.0)	36.4 (10.9–69.2)
	3^rd^	5.1 (0.6–17.3)	74.4 (57.9–87.0)	18.0 (7.5–33.5)
SP	1^st^	100.0 (39.8–100.0)	100.0 (39.8–100.0)	25.0 (0.6–80.6)
	2^nd^	95.2 (86.7–99.0)	92.1 (82.4–97.4)	82.5 (70.9–91.0)
	3^rd^	98.6 (95.0–99.8)	88.7 (82.4–93.4)	88.7 (82.4–93.4)
PPV	1^st^	100.0 (2.5–100.0)	100.0 (15.8–100.0)	40.0 (5.3–85.3)
	2^nd^	50.0 (11.8–88.2)	66.7 (38.4–88.2)	26.7 (7.8–55.1)
	3^rd^	50.0 (6.8–93.2)	64.4 (48.8–78.1)	30.4 (13.2–52.9)
NPV	1^st^	80.0 (28.4–99.5)	100.0 (39.8–100.0)	100.0 (2.5–100.0)
	2^nd^	88.2 (78.1–94.8)	98.3 (90.9–100.0)	88.1 (77.1–95.1)
	3^rd^	79.1 (72.4–84.8)	92.7 (86.9–96.4)	79.8 (72.6–85.7)
*OR*	1^st^	9.0 (0.2–362.8)	45.0 (0.7–3046.0)	2.1 (0.06–77.6)
	2^nd^	7.5 (1.3–43.7)	116.0 (12.23–1101.0)	2.7 (0.7–10.9)
	3^rd^	3.8 (0.5–27.8)	22.8 (9.4–55.5)	1.7 (0.7–4.5)

### Diagnostic performance and parity

The sensitivities for proteinuria which ranged from 23–33% and macrohematuria 8–14% were consistently low in all parity groups (Table 7). The specificity (approximately 92.0%) and positive predictive value (75.0%) of microhematuria was similar among the primigravid and secundigravid women. The overall diagnostic performance of microhematuria (OR = 120.0) and proteinuria (OR = 5.7) was best in secundigravidae.

**Table 7:** Effect of parity on diagnostic potential of urinary indicators of urogenital schistosomiasis.

Diagnostic	Parity	Diagnostic indicators (%)		
parameters		Macrohematuria	Microhematuria	Proteinuria
SS	Primigravidae	14.3 (3.0–36.3)	85.7 (63.7–97.0)	23.8 (8.2–47.2)
	Secundigravidae	8.3 (0.2–38.5)	90.0 (55.5–99.8)	33.3 (9.9–65.1)
	Multigravidae	10.5 (1.3–33.1)	68.4 (43.5–87.4)	26.3 (9.1–51.2)
SP	Primigravidae	97.2 (90.3–99.7)	91.7 (82.7–96.9)	80.6 (69.5–88.9)
	Secundigravidae	97.3 (85.8–99.3)	91.9 (78.1–98.3)	91.9 (78.1–98.3)
	Multigravidae	99.0 (94.6–100.0)	88.0 (80.0–93.6)	88.0 (80.0–93.6)
PPV	Primigravidae	60.0 (14.7–94.7)	75.0 (53.3–90.2)	26.3 (9.1–51.2)
	Secundigravidae	50.0 (1.3–98.7)	75.0 (42.8–94.5)	57.1 (18.4–90.1)
	Multigravidae	66.7 (9.4–99.2)	52.0 (31.3–72.2)	29.4 (10.3–56.0)
NPV	Primigravidae	79.6 (69.6–87.4)	95.7 (87.8–99.1)	78.4 (67.3–87.1)
	Secundigravidae	76.6 (62.0–87.7)	97.1 (85.1–99.9)	81.0 (65.9–91.4)
	Multigravidae	85.3 (77.6–91.2)	93.6 (86.6–97.6)	86.3 (78.0–92.3)
*OR*	Primigravidae	5.8 (0.9–37.6)	66.0 (15.0–290.2)	1.3 (0.4–4.1)
	Secundigravidae	3.3 (0.2–56.8)	102.0 (9.4–1102.0)	5.7 (1.1–30.5)
	Multigravidae	11.7 (1.0–135.7)	15.9 (5.1–49.7)	2.6 (0.8–8.6)

## Discussion

Epidemiological studies abound on urogenital schistosomiasis in sub-Saharan Africa. Little is however known about maternal schistosomiasis in the region. While efforts have recently been made to address this issue in pregnant women [8,9,11,12,31], there are still more to be done for effective management of the disease in the group. Quick detection of the disease in pregnant women is necessary to avert the associated morbidities, which could cause impairment in pregnancy outcomes. Simple reagent strips, often used to monitor medical conditions of pregnant women during antenatal care, could become a very useful tool for quick diagnosis of schistosomiasis for rational control measure.

Urogenital schistosomiasis is a public health problem among pregnant women in the study area. The influence of pregnancy characteristics (i.e. age, trimester, and parity), lack of potable water and socio-cultural belief in regards to transmission had been widely discussed [9,31]. Urogenital schistosomiasis has long been considered as one of the causes of hematuria in endemic communities [25,28]. *Schistosoma-*associated hematuria results from bladder lesions that result from the deposition of the parasite’s spiny eggs in the submucosa [33]. The lack of significant association between hematuria and ages of the pregnant women showed equal predisposition to the condition irrespective of age.

Macrohematuria (visible blood in urine) is used as an indirect rapid diagnostic marker for urogenital schistosomiasis in resource poor rural settings without the availability of urine reagent strips [34–36]. Therefore, the 98.1% specificity observed in this study is consistent with the findings of the aforementioned studies. However, most of those studies were performed on school-based cohorts. The sensitivity of macrohematuria in our study was lower than the mostly reported values in school children [4] but higher than that of a study on preschool children and infants [6]. Gross hematuria increased with increase burden of *S*. *haematobium* infection; an observation similar to previous reports [37,38]. The association reported between infection due to *S*. *haematobium* and microhematuria suggests that it is a good biomarker for infection in pregnant women in endemic areas. This is evident in the high sensitivity and specificity values obtained in our study. Other possible causes of microhematuria which could have led to some false positive results have been suggested to be idiopathic hematuria; peculiar changes in the urogenital tract induced by hormonal and mechanical factors of pregnancy [39,40] and urinary tract infections [41].

The lack of associations between occurrence of proteinuria in *S*. *haematobium* infected and non-infected pregnant women is similar to a report among women of child-bearing age in Tanzania [25]. Aside the various set backs undermining the use of proteinuria and other indirect diagnostic indicators of urogenital schistosomiasis in women, some of which include genitourinary infection and/or the sequelae of genital mutilation [25] and preeclampsia could become a major concern in false positive proteinuria outcomes in pregnant women. Preeclampsia which is often characterized by significant proteinuria during pregnancy is multifactorial including cerebral vasoconstriction, ischemia and vasogenicedema [42], thus a suspected urogenital schistosomiasis-induced proteinuria by dipsticks in pregnant women must be confirmed by microscopy. Occurrence of genitourinary infection and preeclampsia could have resulted in the very poor sensitivity and positive predictive values of proteinuria obtained in our study. High false positivity of proteinuria could have serious implication for preeclampsia management in schistosomiasis endemic areas as the pregnant women may be deprived of the right attention.

The diagnostic performance of reagent strips was greatly influenced by ages of the pregnant women. However, a deviation from the general age-related diagnostic performance in ≥ 30 years needs to be further explored. With maximum sensitivity (100%) and a very high specificity (96.3%) of microhematuria in 15–19 years pregnant women, reagent strips become a very useful tool for diagnosis of *S*. *haematobium* in the group. This success could be attributed to higher *S*. *haematobium* burden in term of intensity of infection in the group. However, more studies are required to ascertain this. Accuracy of reagent strips results had also been reported to be influenced by age and sex of children [43]. Early detection of infection in first trimester can be suggested owning to improved diagnostic performance in the group. This better performance in the group can be related to the reason claimed for the younger pregnant women as earlier mentioned. However, the low number of first trimester participants poses a limitation and thus necessitates further studies. The higher urogenital schistosomiasis detection rate in women in their early trimester which was hypothesized to be related to a lesser frequency of micturition compared to the late trimester women [9] could facilitate easy detection of pathology indicators of the disease. This also could have serious implication on control as disease could progress chronic in nature in late trimesters especially where there is no repeated or fresh exposure to schistosome parasites. Poor performance of hematuria in late trimester however could be due to lower burden of *S*. *haematobium* in term of intensity as shown in our result. While higher parasite burden could be responsible for the better diagnostic performance of microhematuria in primigravidae.

One limitation in this study was the fact that other confounders such as urinary tract infections, idiopathic hematuria or preeclampsia which could also be associated with these urine biomarkers were not considered in the study design. Observations inferred from this study may not be applicable to other population settings. The cross-sectional nature of our study failed to assess variations in presentation of the disease markers with time, thus might have influenced diagnostic performance. Evaluation of macrohaematuria repeatedly over a period of a few days is expected to increase its accuracy [4]. A single urine sample collected and the use of centrifugation method to concentrate parasites rather than the usually recommended filtration method could result in under-presentation of true infection status.

## Conclusion

We observed one fifth of the pregnant women to be infected with *S*. *haematobium* in Yewa North LGA, Ogun State, Nigeria. Microhematuria becomes a useful rapid diagnostic tool for the disease in young pregnant women. However because microhematuria is a condition that occurs during pregnancy and in several other diseases, it is necessary to compliment the diagnosis with other diagnostic tools such as microscopy and serology. Diagnosis of schistosomiasis during early pregnancy stage is recommended and this should be introduced to routine antenatal care in *Schistosoma-*endemic areas. Pregnant women at the later stage of pregnancy should only be treated with praziquantel after confirmatory tests are conducted in order to forestall *S*. *haematobium*-induced adverse pregnancy outcomes.
